# Signaling pathway alterations in hearts of a porcine model harboring a β-myosin heavy chain (MYH7-R403Q) gene variant^☆^

**DOI:** 10.1016/j.jmccpl.2025.100495

**Published:** 2025-11-04

**Authors:** Chad M. Warren, David M. Ryba, Gail E. Geist, Aileen Castro Coronado, Beata M. Wolska, Paul H. Goldspink, R. John Solaro

**Affiliations:** a Department of Physiology and Biophysics, Center for Cardiovascular Research, University of Illinois Chicago, USA; b Bristol Myers Squibb, Brisbane, California, USA; c Department of Medicine, Division of Cardiology, University of Illinois Chicago, USA

**Keywords:** Proteomics, Hypertrophic cardiomyopathy, Fibrosis, Mechano-signaling, Inflammation, Metabolic dysfunction

## Abstract

The disease-causing myosin variant (MYH7-403Q) is linked to hypertrophic cardiomyopathy (HCM). We carried out a research study of signaling pathways in heart samples from control wild-type (WT) GE Yucatán mini-pigs and their littermates harboring the gene variant, MYH7-R403Q. Our approach permits the determination of adverse signaling pathways involved in different regions of a translationally relevant heart without the effects of intervention. We examined the left ventricular free wall (LV), endocardium (EN), and coronary arteries (CA) from 5 transgenic and 5 wild-type mini-pig littermates to determine alterations in global phosphorylation and protein abundance. Digested peptides from 6 to 7 months old mixed-sex mini-pigs were isobarically labeled; 95 % were phospho-enriched, and 5 % were used as the unmodified (total) fraction. The phospho-enriched and unmodified fractions were injected into an Orbitrap Fusion Lumos and analyzed using PEAKS Studio and Ingenuity Pathway Analysis. Surprisingly, we found no significant changes in the phospho-peptide and unmodified protein abundances in CA. Compared to WT, both LV and EN samples displayed minor changes in phosphorylation and significant changes in unmodified proteins. Bioinformatic analysis revealed that pathways associated with mechano-signaling between cardiomyocytes and the extracellular matrix and inflammation were altered in LV and EN samples. In addition, EN samples had larger differences in pathways related to metabolic dysfunction compared to LV. Our findings provide a translational understanding of signaling pathways altered in the MYH7-R403Q gene variant.

## Graphical abstract

**Figure:**
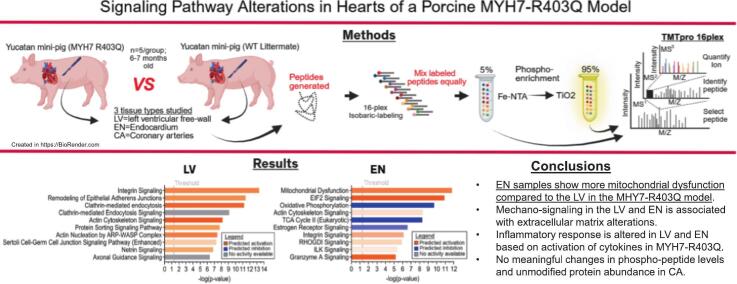

## 1 Introduction

Triggering of hypertrophic cardiomyopathy (HCM) linked to mutations associated with the development of the disease is thought to occur with an increase in myofilament Ca^2+^-sensitivity, inducing a persistent increase in contractility by strongly bound actomyosin cross-bridge interactions [1]. Modulation of Ca^2+−^sensitivity by the development of cardiac myosin inhibitors as a therapeutic approach has been demonstrated to improve the hyper-contractility, ventricular stiffness, outflow obstruction, and depressed exertional tolerance [2]. Cardiac myosin inhibitors were the first sarcomere modulators to receive regulatory approval for the treatment of obstructive HCM [3]. Despite these promising advances, there remains a need for further understanding of the signaling events activated by the mechanical stresses associated with mutant forms of sarcomere and cytoskeletal proteins in HCM. A rationale for this need has been emphasized by demonstrations of the complexity of HCM progression involving multiple regulatory mechanisms in the myocyte and non-myocyte micro-environment [[4], [5], [6]].

Although the mass of cardiac myocytes outweighs other cell types in the heart, they are greatly outnumbered by non-myocyte cells, which are known to participate in hypertrophic signaling, fibrosis, immune activation, inflammation, micro-circulatory dysfunction, vascular and lymphatic endothelial alterations, and sympathetic neuronal alterations [5,7]. Chaffin et al. [5] exemplified this complexity by profiling single nuclei in the left ventricular free wall (LV) from non-failing (NF), HCM, and dilated cardiomyopathy (DCM) human heart samples. The data confirmed transcriptional alterations in the non-myocyte cell populations as described above. Garmany et al. [8] performed multi-omics investigations of human HCM, carried out in controls and tissue obtained from septal myectomies. These results also confirmed the complexity of altered signaling in non-myocyte cells, but the transcriptional profiles demonstrated divergent pathway alterations from those reported in the Chaffin et al. [5] investigation, which may be reflective of disease stage and tissue procurement. Likewise, other profiling studies, for example, demonstrating metabolic dysfunction in HCM, were also carried out in septal samples [9,10].

In addition to understanding the complexity of the cellular constituents of the micro-environment, the extracellular components have also been demonstrated to play an important role in HCM pathology. An earlier study by Sewanan et al. [11] also employed porcine MYH7-R403Q genetically engineered cardiac tissue, and while they did not analyze alterations in signaling, their results underscore the need to understand the signaling events in situ. Sewanan et al. [11] reported that when control induced pluripotent stem cell-derived myocytes were grown on decellularized tissue slices containing HCM extracellular matrix, an HCM phenotype was induced in the myocytes. This induction did not occur on ECM derived from decellularized control tissue slices. These in vitro results highlight the necessity to investigate the complex pathology of HCM within the micro-environment cells in situ without complications from therapies and interventions.

Here, we explored proteomic changes in heart tissue samples from the micro-environment containing both myocyte and non-myocyte cells in control wild-type genetically engineered (GE) Yucatan pigs and GE Yucatan pigs harboring the MYH7-R403Q variant, a common myosin heavy chain mutation that induces HCM [12]. Porcine models are well-documented as appropriate analogs for the human heart due to their functional and anatomical similarities in cardiac and vascular systems. Additionally, proteomic comparisons between human and pig hearts show a close similarity across chambers [13].

We investigated signaling pathways in tissue samples taken from the left ventricular free wall (LV), endocardial (EN), and coronary artery (CA) tissues from 6 to 7-month-old mixed-sex GE Yucatan pigs using unbiased proteomics. In both the LV and EN samples, we found there were significant differences in the unmodified protein abundance in the hearts compared to controls. In contrast, the CA samples did not show significant alterations. Bioinformatic predictions of the canonical signaling pathways associated with the significantly altered proteins demonstrated prominent roles for mechano-signaling between myocytes and the extracellular matrix (ECM), along with an altered immune/inflammatory response in LV and EN samples. However, there were distinctions between predicted pathways in LV and EN samples, with the EN demonstrating a more prominent role in mitochondrial dysfunction. Our findings provide new insights into the signaling hierarchy of the MYH7-R403Q variant and highlight a novel contribution of endocardial dysfunction in disease progression.

## 2 Methods

### 2.1 Yucatán mini-pig tissues

**Fig. 1:**
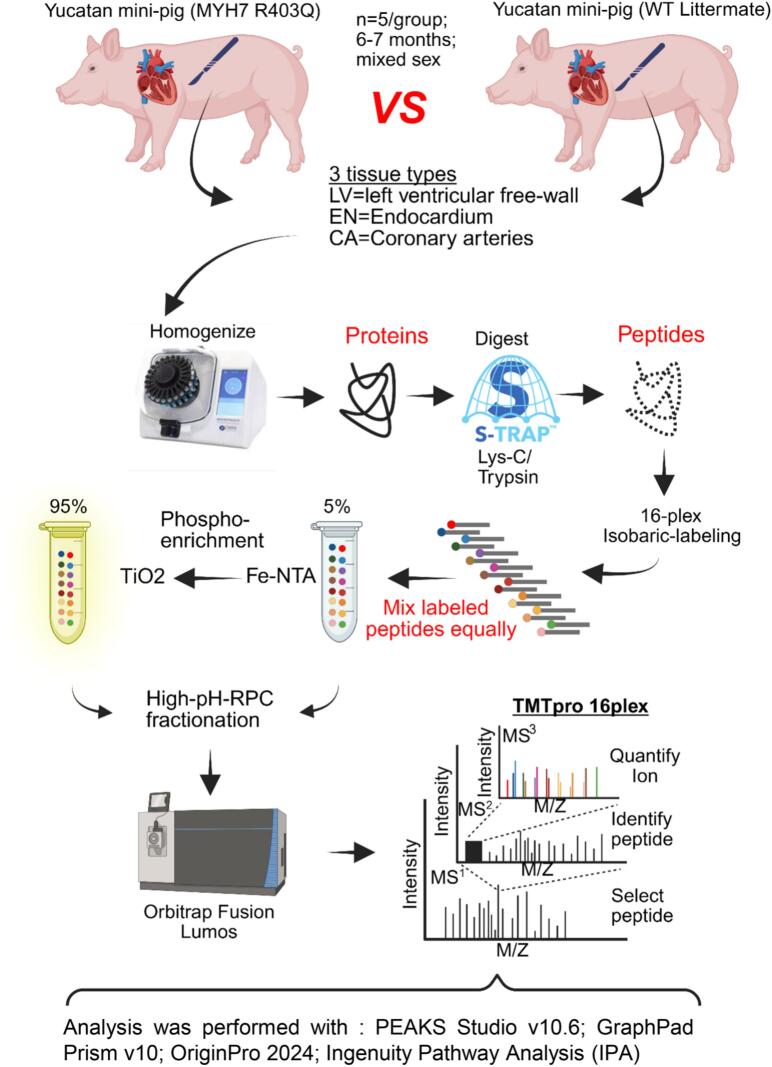
Workflow diagram of the phospho-proteomic approach. Three tissue types from diseased (MYH7-R403Q) and wildtype (WT) Yucatán mini-pig hearts were investigated. The tissues were homogenized with a Bead Ruptor Elite, and the resulting proteins were digested and isobarically labeled. Five percent of the labeled and equally mixed peptides were further fractionated via high-pH-reverse phase chromatography (RPC). At the same time, the remaining 95 % was serially phospho-enriched with iron-NTA and then TiO2. The phospho-enriched peptides were further fractionated via high-pH-RPC, and all fractions were then injected into an Orbitrap Fusion Lumos using synchronous precursor selection. Figure created in https://Biorender.com.

All animal breeding, care, and experiments were performed under and approved by the Institutional Animal Care and Use Protocol at Exemplar Genetics, Sioux Center, IA. Three tissue types were examined: left ventricle free wall (LV), the endocardium of the LV excluding the papillary muscle (EN), and the main left anterior descending coronary artery (CA) were harvested from mutant (MYH7-R403Q) [14] and binarily compared to the wildtype (WT) Yucatán mini-pigs (Fig. 1). Animals were fasted for a minimum of 12 h (overnight) before being initially sedated with an IM injection of tiletamine/xylazine mixture in sterile saline in their home pen. Once the animal was sedated, it was moved to a procedure room and placed on a nose cone supplying 2.5–3 % isoflurane in 100 % oxygen at 1–2 L/min. IV access was obtained via either the lateral or medial auricular vein. Animals were intubated in sternal recumbency with an endotracheal tube of appropriate size (6.0–7.0 mm), and mechanical volume-controlled positive pressure ventilation was initiated (8–10 mL/kg, 10–15 bpm) with 100 % oxygen and isoflurane concentration maintained at 2.5–3 %. The animal was repositioned to right lateral recumbency. A butorphanol tartrate constant rate infusion was then initiated via the auricular vein at 0.1–0.3 mg/kg/h IV and a left-sided paraspinal block in the region of ribs 2–4 was established with 0.25–0.5 % bupivacaine HCl (approximately 2 mL at each rib space). A left lateral thoracotomy was then performed at the 4th intercostal space, and the ascending aorta was isolated. 100 U/kg heparin sodium was administered IV via the auricular vein and allowed to perfuse before the animal was euthanized by rapid IV administration of 2 mEq/kg KCl IV. The heart was then rapidly excised and retrogradely perfused with ice-cold Custodiol® cardioplegia solution, and tissues were collected and immediately flash frozen in liquid nitrogen. The animals were 6–7 months old, which is about the age of sexual maturity [15], corresponding to the age in humans of about 8–13 years, and there were 2 females and 3 males in each group. The phospho-proteomic approach used a biological *n* = 5 in each group for a total of 30 samples.

### 2.2 Phospho-proteomic sample preparation

The tissue (12–18 mg) was homogenized in 8 M Urea, 50 mM triethylammonium bicarbonate (TEAB) pH 8.5, 2 mM MgCl_2_, 10 mM Tris(2-carboxyethyl)phosphine hydrochloride (TCEP), 100 nM calyculin A, 1:100 *v*/v phosphatase inhibitor (Millipore # 524624) at a 1:15 tissue: buffer *w*/*v* ratio. The homogenization was performed with a Bead Ruptor Elite (Omni International) in 0.5 mL tubes with 1.4 mm ceramic beads set at power 5 m/s, 30 s, 3 cycles with 3 min dwell between cycles. After the homogenization, 20 % (*w*/*v*) SDS was added to a final 5 % SDS concentration and shaken with a vortexer for 10 min, then sonicated in a water bath for 10 min. The samples were then centrifuged and clarified at 14,000 X g for 5 min at room temperature. The protein concentration was determined with the Pierce 660 nm protein assay (#22660), including the ionic detergent compatibility reagent (#22663), following the manufacturer's protocol. All samples were diluted to 2 mg/mL with lysis buffer and 5 % (*w*/*v*) SDS.

### 2.3 Immunoblotting

Refer to the supplementary methods file.

### 2.4 Protein S-TRAP digestion and TMTpro labeling

Refer to the supplementary methods file.

### 2.5 Phospho-peptide enrichment and high pH reverse-phase fractionation

Refer to the supplementary methods file.

### 2.6 Mass spectrometric data acquisition

Refer to the supplementary methods file.

### 2.7 Mass spectrometric database alignment and TMT quantification

Refer to the supplementary methods file.

### 2.8 Statistical analysis

The immunoblot images were densitometrically analyzed with BioRad's ImageLab v. 6.1 and Microsoft Excel 360. The data were statistically analyzed and graphed with GraphPad Prism v. 10.5. Outliers were identified with a ROUT test set at Q = 5 %. Data were analyzed by a two-tailed unpaired *t*-test with normally distributed data (all data were normal based on D'Agostino-Pearson omnibus (K2)) and similar variances. When the variances were statistically different via an F-test, *p* < 0.05, a Welch's *t*-test was applied. All data are reported as mean ± standard deviation, with p < 0.05 considered significant, biological *n* = 4–5 with no technical replicates.

Mass spectrometry data were exported from PEAKS Studio and imported into GraphPad Prism 9.5.1 to Log2 transform the data and use an unpaired *t*-test with an individual variance for each row assumption. Multiple comparisons were calculated to determine the false discovery rate utilizing a two-stage linear step-up method [16] with a Q-value and *P*-value set at 0.05 for significance. The data distribution was analyzed in GraphPad Prism by Skewness and Kurtosis, which indicated a normal distribution in all data sets. The intensity data were imported into OriginPro 2023b (64-bit) for principal component analysis (PCA).

### 2.9 Bioinformatics

The proteomic data containing gene names, UniProt identifiers, Log2 ratios, *P*-values (amino acid position numbers when analyzing phospho-peptide data), and Q-values were imported into Ingenuity Pathway Analysis (IPA) for core analysis [17]. The core analysis was set to determine indirect and direct relationships between genes and endogenous chemicals based on experimental log ratio, *p*-value, and false discovery rate (q-value). IPA generates probable canonical and upstream regulator pathways via a right-tailed Fisher's exact test to calculate a *P*-value to explain the association between our dataset and the theoretical dataset. Complete proteomic datasets were imported separately for LV and EN binary comparisons in IPA, and a ≥ 20 % difference cutoff was applied along with a *p*-value and q-value ≤0.05 for the LV and a ≤ 0.02 p-value cutoff for EN.

## 3 Results and discussion

### 3.1 Translational phospho-proteomic approach interrogating the myocyte micro-environment

The genetically engineered MYH7-R403Q Yucatán mini-pig model is a well-established translational model [14,[18], [19], [20]]. The MYH7-R403Q transcript expression levels were previously determined to be in a ratio of ∼1:1 with the WT [18]. This same report by Anderson et al. also reported semi-quantitative mass spectrometry data that also confirmed a ratio of ∼1:1 at the protein level [18]. The MYH7-R403Q Yucatán mini-pig model has been reported to have an enhanced myofilament response to Ca^2+^ in isolated fibers, as well as in vivo diastolic dysfunction with elevated filling pressures and limited cardiac reserve [19]. This sarcomeric point mutation was the first HCM (previously known as familial hypertrophic cardiomyopathy) mutation discovered [12] and has been studied extensively. However, to our knowledge, no global phospho-proteomic investigation has been conducted on this mini-pig model. Previous studies have investigated the same Yucatan mini-pig model used in the present study at 3 months of age and reported the presence of fiber disarray and interstitial fibrosis [11]. Sewanan et al. [11] also noted evidence of tissue remodeling and increased passive tension, consistent with HCM pathology.

Our investigation aimed to discover if the model demonstrates altered signaling pathways by mechanisms involving changes in global phosphorylation and unmodified protein abundance in different regions of the heart compared to controls. Importantly, our data were generated in samples without the interpretative compromises associated with long-term HCM management therapies such as β-adrenergic blockers, Ca-channel blockers, disopyramide, and anti-coagulant prophylaxis [21]. The workflow (Fig. 1) allowed us to simultaneously prepare samples for global protein and phospho-peptide abundance determinations. By using tissue samples comprised of many cell types (myocytes and non-myocytes), as opposed to single-cell derived samples, we were able to investigate the complex micro-environment for protein abundance and phosphorylation alterations. Isobarically labeling 16 samples separately with TMTpro 16-plex and then equally combining them to one multiplexed sample allowed 16 times more peptide (∼1.52 mg) for the serial phospho-enrichment (Fig. 1). Furthermore, the increased starting material used for the enrichment yielded enough phosphorylated peptides to allow further fractionation with high pH reverse phase chromatography to increase the depth of coverage. The refined method and use of a more translationally relevant model utilizing Yucatán mini-pigs permitted global phospho-proteomic mass spectrometry interrogation.

**Fig. 2:**
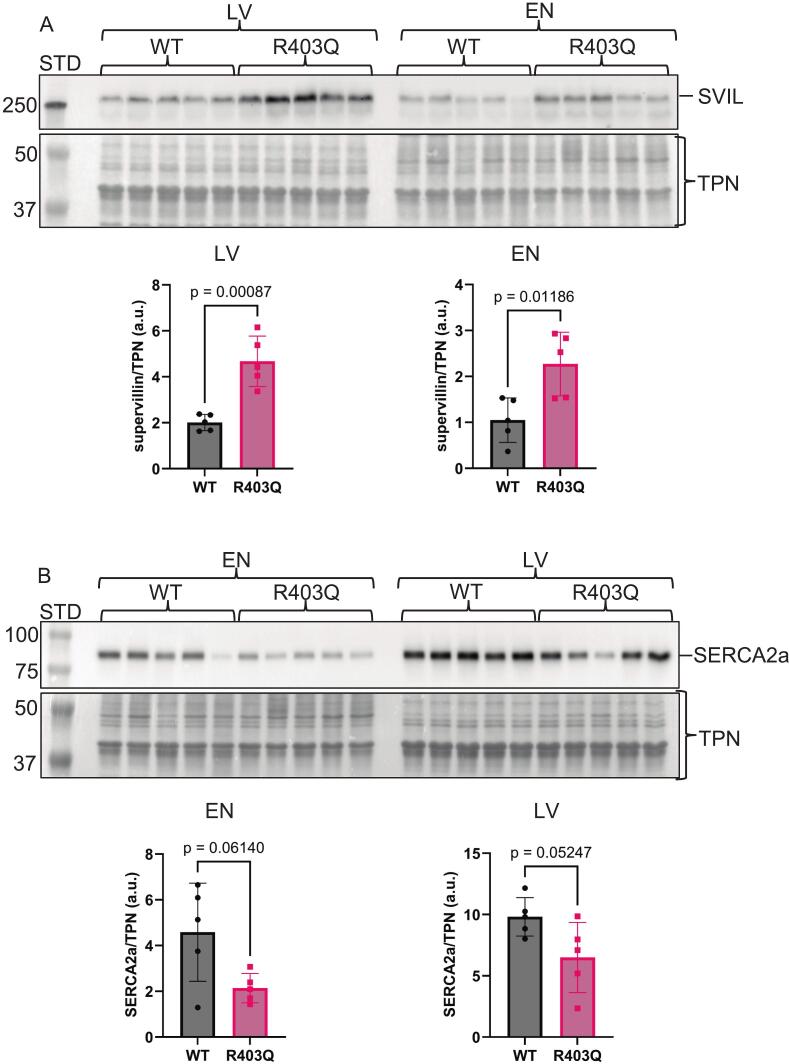
Immunoblot analysis of known hypertrophic cardiomyopathy protein markers. A. Cropped Western blot image of supervillin (SVIL) on top and below a cropped total protein-stained image (Swift™ stain) of the membrane used for total protein normalization (TPN). The quantitation is shown below the images as a scatter dot plot. B. Cropped Western blot image of sarcoplasmic/endoplasmic reticulum Ca^2+^ ATPase 2a (SERCA2a) on top and below a cropped total protein-stained image (Swift™ stain) of the membrane used for total protein normalization (TPN). The quantitation is shown below the images as a scatter dot plot. Data were analyzed by a two-tailed unpaired *t*-test with normally distributed data and similar standard deviations. Exact *p*-values are shown on scatter dot plots, with *p* < 0.05 considered significant. The only exception is the SERCA2a EN group comparison, where the standard deviations were different and thus a Welch's t-test was applied. All data are reported as mean ± standard deviation, biological *n* = 5 separate animal hearts per group, with no technical repetition. STD, molecular weight standard; LV, left ventricle; EN, endocardium; WT, wildtype; R403Q, MYH7-R403Q mutant. Note: the membrane used to probe SERCA2a was also used to probe for alpha-actinin in Fig. 6, so the swift-stained images are the same.

**Fig. 3:**
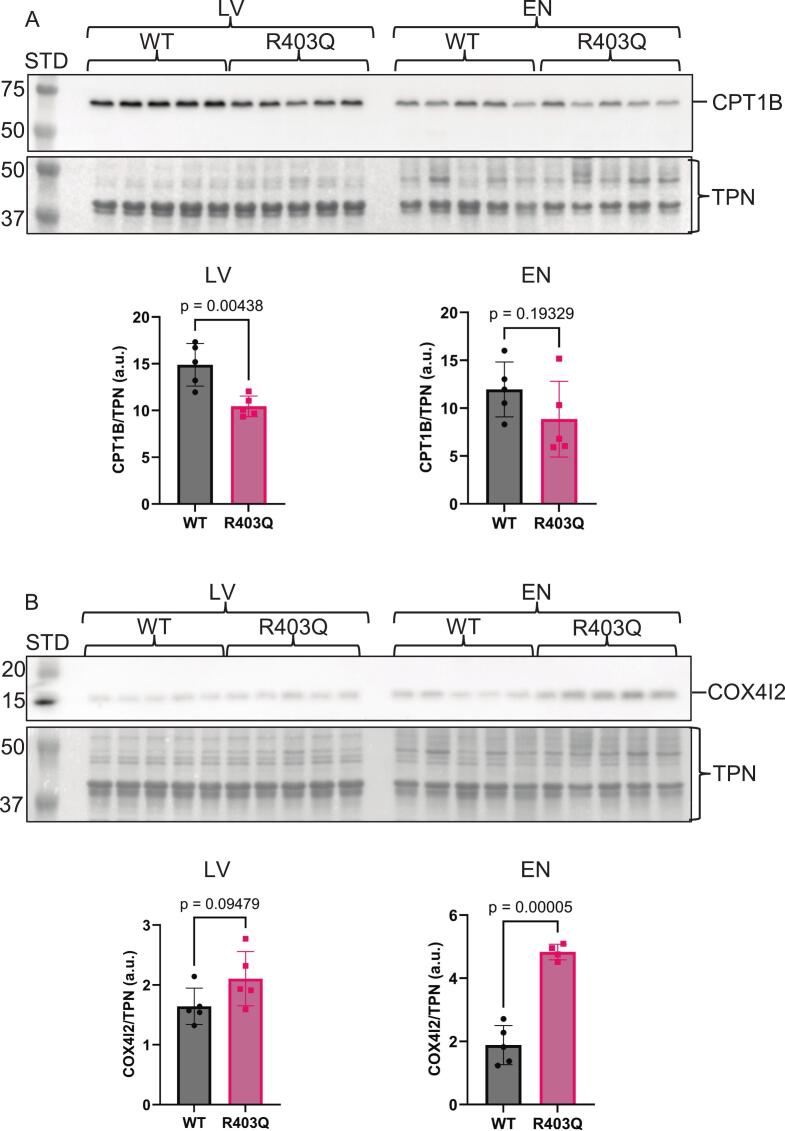
Immunoblot analysis of known mitochondrial and hypertrophic cardiomyopathy protein markers. A. Cropped Western blot image of camitine palmitoyltransferases 1B (CPT1B) on top and below a cropped total protein stained image (Swift™ stain) of the membrane used for total protein normalization (TPN). The quantitation is shown below the images as a scatter dot plot. B. Cropped Western blot image of cytochrome *c* oxidase 4 isoform 2 (COX1I2) on top and below a cropped total protein stained image (Swift™ stain) of the membrane used for total protein normalization (TPN). The quantitation is shown below the images as a scatter dot plot. Data were analyzed by a two-tailed unpaired *t*-test with normally distributed data and similar standard deviations. Exact *p*-values are shown on scatter dot plots, with p < 0.05 considered significant. All data are reported as mean ± standard deviation, biological *n* = 5 separate animal hearts per group, with no technical repetition, except for the COX4I2 EN R403Q group, which had a biological *n* = 4. STD, molecular weight standard; LV, left ventricle; EN, endocardium; WT, wildtype; R403Q, MYH7-R403Q mutant. Note: the membrane used to probe COX4I2 was also used to probe for vinculin in Fig. 6, so the swift-stained images are the same.

Even though this Yucatán mini-pig model is well-established, as mentioned above, upon gross examination during tissue dissection of the hearts used here, thickening of the endocardial layer and the left ventricular free wall was clearly altered in the hearts compared to the WT controls (personal communication). A disease phenotype was indirectly supported via immunoblotting for several known proteins associated with HCM (Fig. 2, Fig. 3A, Supplementary Fig. S1, Supplementary Fig. S2 A-B). The actin-binding protein supervillin abundance was significantly increased in both the LV and EN HCM mutant samples (Fig. 2A, Supplementary Fig. S1A—B) and is a known biomarker for HCM [22,23]. The Ca^2+^ transporter critical for heart contraction, sarco/endoplasmic reticulum Ca^2+^-ATPASE 2a (SERCA2a), was trending downward (Fig. 2B, Supplementary Fig. S1C—D) and is linked to HCM [24,25]. An essential enzyme for fatty acid oxidation, carnitine palmitoyltransferase 1B (CPT1B), is also known to be downregulated in HCM [26] was significantly reduced in the LV and trending down in the EN samples (Fig. 3A, Supplementary Fig. S2A—B). As an orthogonal approach, our immunoblot data corroborate the mass spectrometry data (Supplementary Table S1) and indirectly support the pathology of our genetically engineered MYH7-R403Q Yucatán mini-pig model.

### 3.2 Mass spectrometry and phospho-peptide analysis

The number of unmodified total proteins identified in the CA groups was 6028, with 2192 protein groups (Supplementary Table S1). Despite this robust analysis, unexpectedly, the CA group did not have any meaningfully altered unmodified total proteins (Supplementary Fig. S3B) between the WT and R403Q groups based on a q-value <0.05 (FDR). Clinical interrogation of adults with advanced HCM shows that 30–50 % of these patients demonstrate CA disease [27,28]. Micro-vasculopathy has been described histologically in advanced HCM, but its impact and presence are not clearly understood [29]. Our finding of no differences between the WT and R403Q groups may be related to the fact that we collected samples from the large CA.

**Fig. 4:**
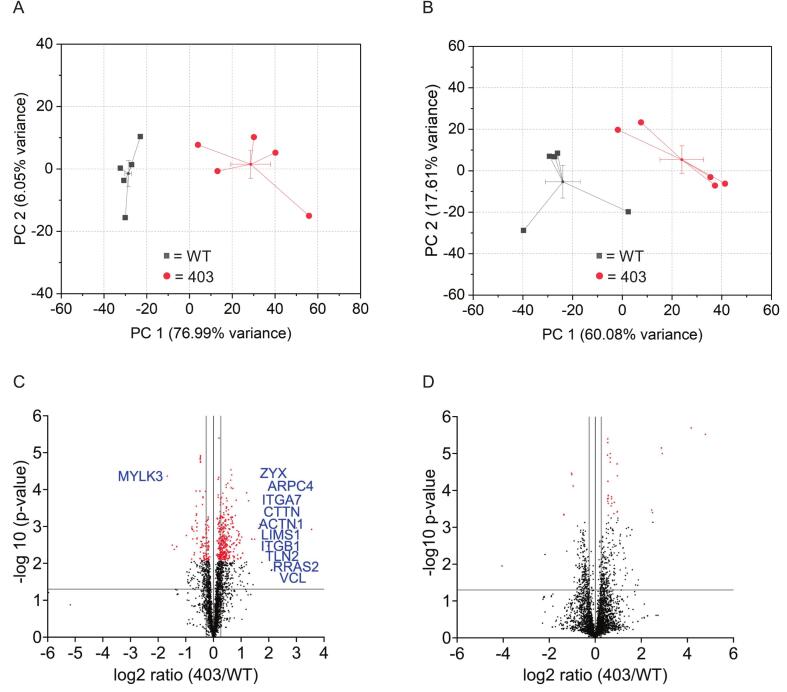
Unmodified total proteomic analysis of data distribution and differences in left ventricle free-wall (LV) and endocardium (EN) samples. A. Principal component (PC) analysis of total unmodified proteins of LV shows two distinct populations for the wild type (WT) compared to the MYH7-R403Q mutant (403). Error bars represent standard error, *n* = 5. B. Principal component (PC) analysis of total unmodified proteins of EN shows two distinct populations for the WT compared to 403. Error bars represent standard error, n = 5. C. Volcano plot of differential protein abundances of total unmodified proteins of LV between the 403 and WT, plotting -log10 *p*-values and log2 ratio, n = 5. Vertical gray lines = ≥ ±20 % difference; horizontal gray line multiple unpaired *t*-test <0.05 p-value. Note: red points have a q-value <0.05 (FDR) based on multiple comparison corrections with a two-stage step-up (Benjamini, Krieger, and Yekutieli). The protein gene callouts are integrin and actin cytoskeleton signaling molecules. D. Volcano plot of differential protein abundances of total unmodified proteins of EN between the 403 and WT, plotting -log10 p-values and log2 ratio, biological *n* = 5 separate animal hearts per group, with no technical repetition. Vertical gray lines = ≥ ±20 % difference; horizontal gray line multiple unpaired t-test <0.05 p-value. Note: red points have a q-value <0.05 (FDR) based on multiple comparison corrections with a two-stage step-up (Benjamini, Krieger, and Yekutieli). (For interpretation of the references to colour in this figure legend, the reader is referred to the web version of this article.)

The unmodified protein abundance between mini-pig controls and MYH7-R403Q variant samples was altered in the LV and EN group comparisons. The number of unmodified total proteins identified in the LV group was 5762, with 2164 protein groups; in the EN group, 6115, with 2986 protein groups (Supplementary Table S1). Data distributions analyzed by principal component analysis (PCA) show the wildtype (WT) and the mutant R403Q separated in the LV, EN, and CA groups (Fig. 4A-B, Supplementary Fig. S3A), indicating distinct identities. To display the effects of the MYH7-R403Q variant mutation more clearly, volcano plots of the proteomic datasets from the LV (Fig. 4C) and EN (Fig. 4D) samples comparing the WT and R403Q with the proteins involved in actin cytoskeleton and integrin pathway alterations are shown in the LV group (Fig. 4C).

In addition to the unmodified total proteins, we wanted to consider phosphorylation alterations, and the PCA data distributions of phospho-peptides from the LV, EN, and CA samples (Supplementary Figs. S4A, S4B, S3C). All the phospho-peptides and the statistical results are listed for all groups in Supplementary Table S2. Surprisingly, only two phospho-peptides were meaningfully altered in the R403Q LV and EN pig model: spectrin beta chain (decreased in LV) and LIM domain-binding 3 protein (increased in EN) (Supplementary Figs. S4C, S4D, S3D), and both are associated with the actin cytoskeleton [30,31]. Spectrin beta chain is well known to function as a scaffold protein that links the plasma membrane to the actin cytoskeleton, but it has also been implicated in maintaining normal cardiac electrical activity and mitochondrial function, among other functions [32,33]. LIM domain-binding 3 protein has been associated with human HCM [34] and has a role in mechanosensory actin cytoskeleton remodeling [35]. Indeed, a surprising finding here in the mini-pig MYH7-R403Q variant model was the mutation's lack of impact on levels of mainly serine and threonine protein phosphorylation. Our previous experience and demonstration of proteome and phospho-proteome modifications in other cardiac disorders indicate the reliability of our findings [36,37]. In addition, we were able to identify and quantify 10,873 LV, 11,100 EN, and 14,634 CA phospho-peptides per comparison after filtering (see supplementary methods), which is a comparable sensitivity to other groups [38,39]. However, the biological context is in a large animal model with 5 biological replicates that had variability, which may have contributed to the lack of significance. In contrast, a study investigating tyrosine phosphorylation using a mouse model of R403Q-αMyHC found significant tyrosine phosphorylation changes [40]. The differences in animal models, myosin heavy chain isoforms, and targeted phospho-species may explain the differences.

### 3.3 Effects of MYH7-R403Q variant on the LV free wall

**Fig. 5:**
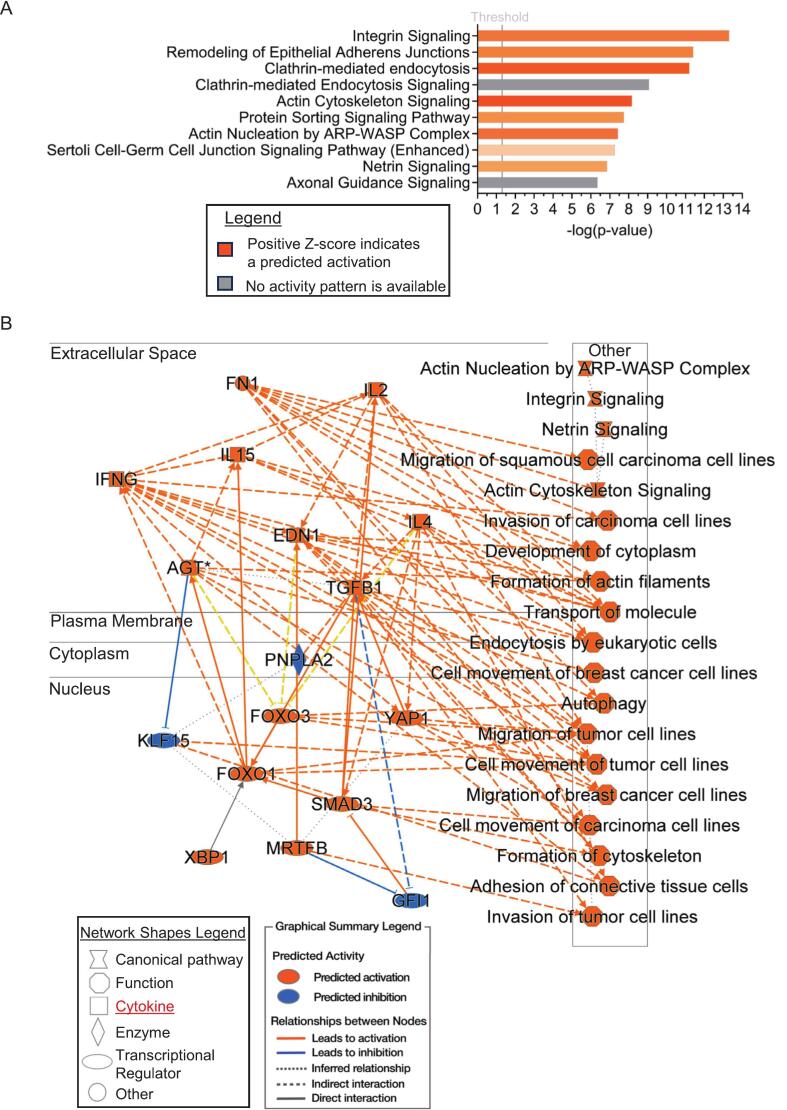
Ingenuity pathway analysis (IPA) of unmodified total proteomic data from left ventricle free-wall (LV) suggests compromised cell-to-cell interactions via the extracellular matrix and an altered inflammatory response in the R403Q animals. A. The top 10 altered canonical pathways were discovered with IPA (right-tailed Fisher's exact test) in the LV. The gray threshold line indicates a p-value of 0.05, indicating the integrin signaling pathway as the most significant, with a predicted activation based on a *Z*-score in IPA. B. IPA graphical summary of the LV data predicting the most alterations in the extracellular space and nucleus. The predicted activation of many cytokines in the extracellular space suggests that inflammation may be contributing to the pathology. Biological n = 5 separate animal hearts per group, with no technical repetition.

IPA data shown in Fig. 5 emphasize the predominant role of mechanical stress in modifying the proteome of the MYH7-R403Q variant mini-pig LV. Our findings indicate that the integrin signaling network is a major process associated with the MYH7-R403Q variant in the mini-pig heart model, supporting our approach of interrogating not only the cardiac myocytes but also their environment. Integrins exist at the cell surface of cardiac myocytes and fibroblasts as heterodimers with a structural role as well as a bidirectional conduit for communication of these cells with the extracellular matrix [41]. Integrin communication between the ECM and cardiac myocytes involves a network of signaling molecules that are proximal to pathways controlling survival, shape, and polarity. These signaling molecules include FAK (focal adhesion complex), ILK (integrin-linked kinase), and PINCH (particularly interesting new cysteine-histidine-rich protein) [42]. These signaling molecules cooperate to promote interactions of integrins with RTKs (receptor tyrosine kinases) to maintain homeostasis. Disturbances of this communication can promote hypertrophic responses [43].

**Fig. 6:**
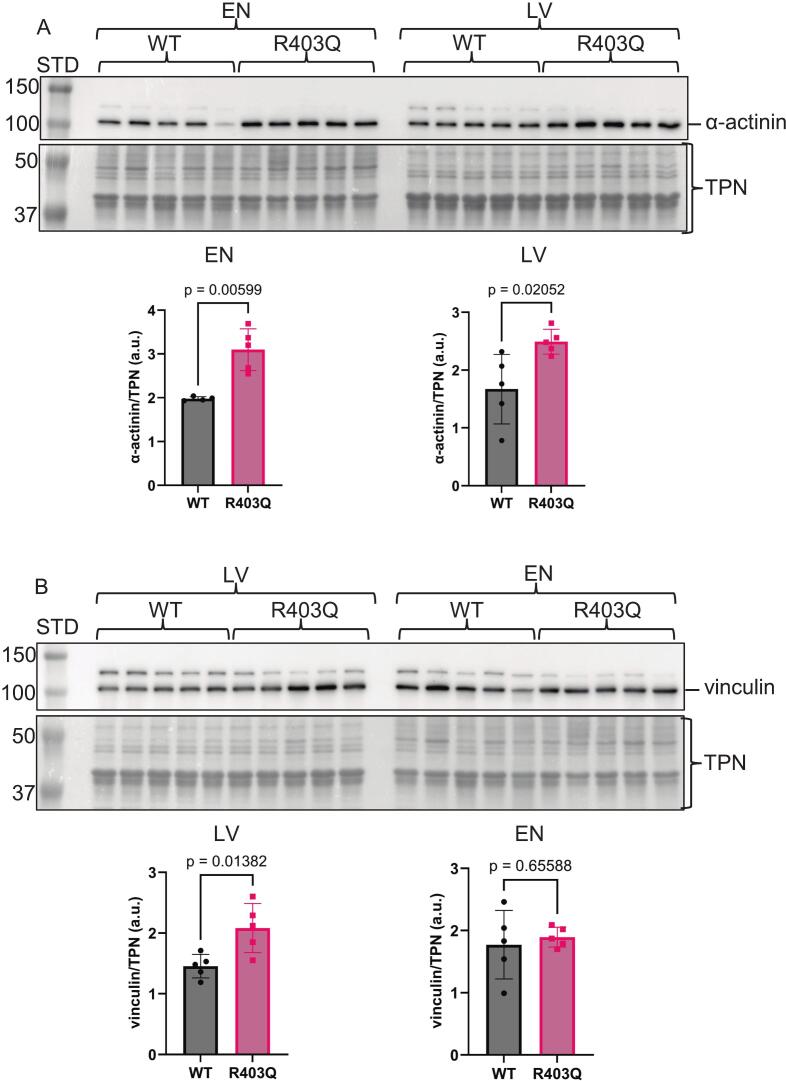
Immunoblot analysis of known cytoskeletal and integrin pathway proteins. A. Cropped Western blot image of alpha-actinin (α-actinin) on top and below a cropped total protein-stained image (Swift™ stain) of the membrane used for total protein normalization (TPN). The quantitation is shown below the images as a scatter dot plot. B. Cropped Western blot image of vinculin on top and below a cropped total protein-stained image (Swift™ stain) of the membrane used for total protein normalization (TPN). The quantitation is shown below the images as a scatter dot plot. The vinculin LV and alpha-actin EN group comparisons were analyzed by a two-tailed unpaired t-test with normally distributed data and similar standard deviations. While the vinculin EN and alpha-actinin LV group comparisons were analyzed by with a Welch's t-test due to dissimilar standard deviations. Exact p-values are shown on scatter dot plots, with *p* < 0.05 considered significant. All data are reported as mean ± standard deviation, n = 5 separate animal hearts per group, with no technical repetition, except for the alpha-actinin LV WT group, which had a biological *n* = 4. STD, molecular weight standard; LV, left ventricle; EN, endocardium; WT, wildtype; R403Q, MYH7-R403Q mutant. Note: the membrane used to probe alpha-actinin and vinculin was also used to probe for SERCA2a and COX4I2, respectively, in Fig. 2, Fig. 3, so the swift stained images are the same.

This network includes integrin-related transmembrane signaling connecting to the sarcomeres and from the sarcomeres that engage downstream signaling coordinated by cytoskeletal and *Z*-disk proteins to hypertrophic signaling proteins such as RAS and AKT. Upregulated integrin-related proteins demonstrated to be important components of this signaling network include ITGA7 (integrin alpha7beta1) and ITGB1 (integrin subunit binding protein 1 [44,45]. Upregulated cytoskeletal network proteins include: TLN2 (talin-2), and VCL (vinculin) [46,47]. Upregulated proteins interacting between the Z-disk and the cytoskeleton include ACTN1 (alpha-actinin 1), ZYX (zyxin), and LIMS1 (LIM zinc finger domain 1) [45,48,49] were all identified and highlighted on the volcano plot in Fig. 4C. An orthogonal immunoblotting approach validated our mass spectrometry results, indicating an increase in alpha-actinin and vinculin, thereby supporting alterations in the integrin signaling network and the broader ECM in the MYH7-R403Q variant (Fig. 6, Supplemental Fig. S5). Fig. 4C also shows upregulation of proteins that have not been reported in the MYH7-R403Q variant. One is the hypertrophic signaling protein RRAS2 (ras-related protein R-Ras2), which facilitates binding of RAS to PI3K and is associated with Noonan Syndrome [50]. Another is the ARPC4 (Arp2/3 complex subunit 4), which regulates the branching of filamentous actin on existing filaments. [51]. As expected, some of our findings reflect altered pathways that are seen in other cardiac disorders. Even though the severity of HCM is higher in sarcomere-linked than non-sarcomeric HCM, mechanical stress induces and plays a role in signaling in both disorders, known to occur in non-sarcomeric HCM and hypertrophy in the adult [52].

Some of our pathway analysis predictions in the LV demonstrate unexplored avenues of signaling in HCM. A top pathway prediction in the LV was the remodeling of epithelial adherens junctions (Fig. 5A). Single-cell transcriptomic studies in human HCM septal samples have demonstrated significant alterations in tight, gap, and adherens junctions [53]. The authors conclude that this modification may serve as an early-stage biomarker for HCM. The single-cell analysis also revealed significant modifications in the actin cytoskeleton and protein sorting, as we report in Fig. 5. Another top finding in the pathway analysis of the LV from porcine hearts with the MYH7-R403Q variant was a significant upregulation of clathrin-mediated endocytosis. This is a new finding, which, to the best of our knowledge, has not been previously reported in HCM. This mechanism does promote hypertrophic signaling; evidence shows that internalization of the apelin G protein-coupled receptor (APJ) is mediated by a mechano-sensitive mechanism, inducing clathrin-mediated endocytosis and hypertrophic signaling [54,55]. Also among the top predictions is the activation of protein sorting pathways in the porcine LV from the MYH7-R403Q variant samples. Protein sorting pathways are critical to homeostatic cellular functions by their action to ensure that newly synthesized proteins are transported to their correct locations within the cell [56]. Our finding of an activation of the pathway in the MYH7-R403Q variant has not been investigated and may represent a direction for future research leading to increased understanding.

The graphical summary of the IPA data from the LV group indicates most of the alterations were present in both the extracellular space and the nucleus, with many cytokines in the extracellular space being activated, suggesting an inflammatory response to the MYH7-R403Q variant (Fig. 5B). Signaling cascades mediated by integrins are critical in the myocardium as documented to be significantly involved in inflammation via interleukins and fibrosis via TGFβ [42]. An example is the evidence that dysfunction signaling via integrin-αVβ6, leading to TGF-β signaling, serves as a driver of cardiac fibrosis [57]. In vivo inhibition of integrin-αVβ6 induced reduced expression of profibrotic markers and reduced fibrosis formation in mouse models of arrhythmogenic cardiomyopathy [57]. Further evidence supports a prominent role of myocyte-ECM signaling, likely to involve integrins and promote HCM. Sewanan et al. [11] reported evidence that an HCM phenotype could be induced in normal iPSC cardiomyocytes grown on decellularized myocardium from a GE Yucatan porcine HCM model (MYH7-R403Q). The data strongly support the hypothesis that fibroblasts from HCM hearts can contribute to disease progression even though the instigating disease gene is disabled.

### 3.4 Comparison of the EN and the LV free wall

**Fig. 7:**
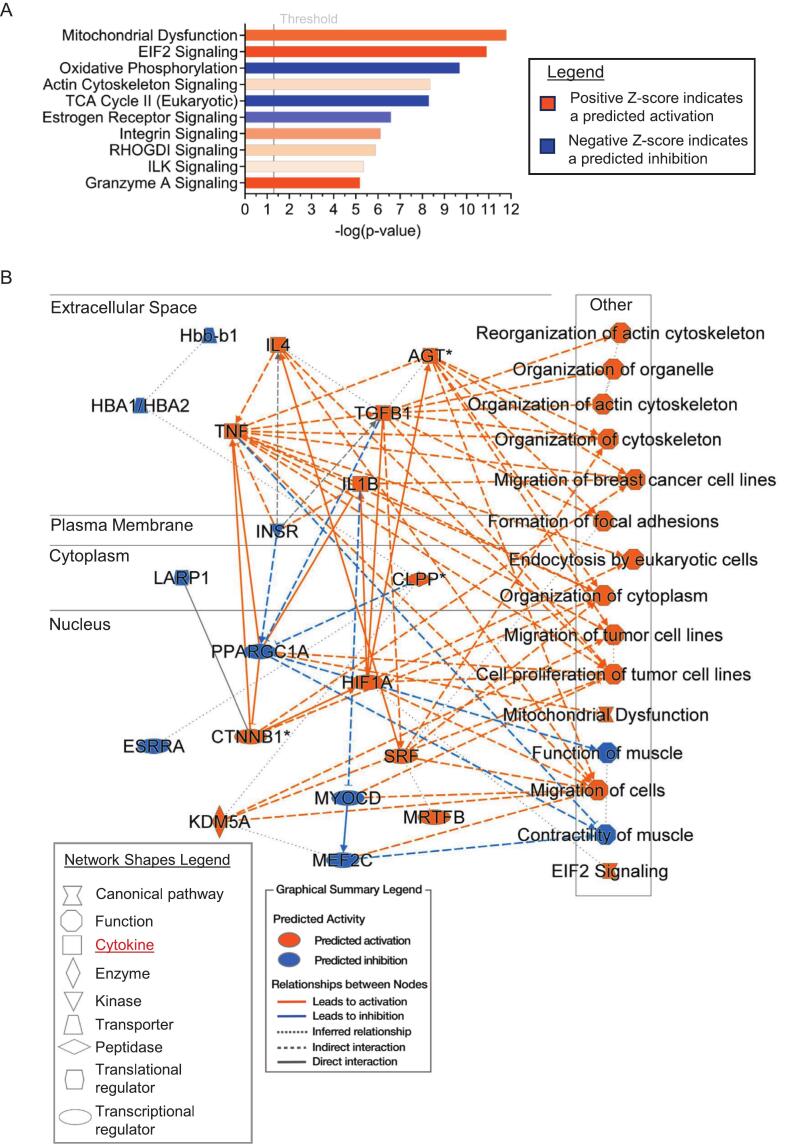
Ingenuity pathway analysis (IPA) of unmodified total proteomic data from endocardium (EN) suggests it compromised cell-to-cell interactions via the extracellular matrix and an altered inflammatory response, but with additional mitochondrial dysfunction in the R403Q animals. A. The top 10 altered canonical pathways were discovered with IPA (right-tailed Fisher's exact test) in the EN. The gray threshold line indicates a p-value of 0.05, indicating the mitochondrial dysfunction pathway as the most significant, with a predicted activation or inhibition based on a Z-score. B. IPA graphical summary of the EN data predicting the most alterations in the extracellular space and nucleus. The predicted activation of many cytokines in the extracellular space suggests that inflammation may be contributing to the pathology. Biological n = 5 separate animal hearts per group, with no technical repetition.

There were clear differences between the LV and EN samples. The EN samples that compared the WT to the R403Q predicted mitochondrial dysfunction as the top-activated pathway, along with the predicted inhibition of oxidative phosphorylation, TCA cycle II, and estrogen receptor (ER) signaling, further suggesting reduced metabolic activity in the EN MYH7-R403Q variant (Fig. 7). In the EN samples, cytochrome-c-oxidase subunit 4 isoform 2 (Cox4I2) was significantly increased based on immunoblotting (Fig. 3B, Supplementary Fig. S2C—D). Cox4I2 regulates the terminal electron acceptor of the oxidative phosphorylation system in the mitochondria, which was predicted to be inhibited in the EN group (Fig. 7A) and has been suggested to be preferentially expressed under oxidative stress as the more efficient isoform [58]. Together with the mass spectrometry data, this provides further evidence of mitochondrial dysfunction in the EN group. In a recent report by Krause et al. [59], which studied the same R403Q mini-pig model as we did, but with only males at 8 months of age and from apex LV tissue, generally agrees with our mitochondrial dysfunction finding in the EN samples. An important distinction was that we used the LV free wall and EN to look at regional differences, while they used the apex of the LV and compared it to the left atrium [59]. Although both EN and LV samples in the MYH7-R403Q variant hearts exhibited altered mechano-signaling pathways and immune/inflammatory responses, alterations in mitochondrial energetics were more evident in EN than in LV samples (Fig. 5, Fig. 7). The EN demonstrated mitochondrial dysfunction and increased inflammatory signaling, which may also be related to the reduced ER signaling. Regulation of gene transcription by ERα and ERβ involves binding to genomic and mitochondrial DNA. A result of this transcriptional activity is that there is stimulation of signaling cascades important in the maintenance of mitochondrial function and morphology [60]. Previous studies identified the expression of ER-β as cardio-protective [61] in pressure overload, hypertrophy was followed by further studies showing that ER signaling is also anti-fibrotic [61,62]. In an investigation of mechanisms involved in ER signaling, it was shown that the effects of angiotensin II (AngII) stress were diminished by the treatment of cellular systems with the ER agonist β-LGND2 [63]. ER signaling involves modulation of the endogenous apelin ligand interacting with the G-protein-coupled apelin receptor. AngII stress induced a reduction in the expression of apelin/apelin receptor signal that is cardio-protective, but the ER agonist overcame this reduction [63]. The ER agonist also depressed the pro-fibrotic response of fibroblasts to AngII by inhibiting the HDAC5 phosphorylation and SMAD activation. In contrast, there is evidence that apelin 13 isoform induces myocyte hypertrophy, oxidative stress, and endoplasmic reticulum stress responses. As with many GPCR signaling cascades, this result suggests an optimal range of apelin for maintaining homeostasis and acting as a cardio-protective ligand. [64]

However, some similarities were presented in the IPA graphical summary of the LV and EN samples, where similar cytokines (TGFB1, IL4, AGT) were activated (Figs. 5B, 7B), indicating a common inflammatory response to the MYH7-R403Q variant in both tissue types. The MYH7-R403Q mutation affects cell-to-cell communication via the ECM and fibronectin-1, which helps form the ECM, and was activated in the LV (Fig. 5). Overall, the LV had a pro-fibrotic program activated in association with the ECM. Although the EN was more affected by mitochondrial dysfunction, both the LV and EN samples had an inflammatory response based on the predicted increase in cytokine activity. An important finding about variable pathological pathways between the LV free wall and EN is that estrogen receptor (ER) signaling was uniquely inhibited in the EN (Fig. 7A). Prominent in the ER signaling cascade is the most powerful estrogen in humans, 17β-estradiol (E2). Although more studies like those reported here need to be carried out, ER signaling inhibition indicates an adverse effect with the possibility of a more severe phenotype in females than males. Increased signaling via granzyme (Gzm) also represents a novel finding in the MYH7-R403Q-related pathway discovery that could inform therapeutic strategies. Our data revealed an upregulation of GzmA signaling in the EN samples (Fig. 7A). Gzms are present in cytotoxic T cells and released in immune processes. Generally, this release and cytotoxic function, which is evident in myocarditis, requires perforin for translocation [65]. However, in the case GzmB, evidence has been reported showing that a deficiency promotes protection in AngII-induced fibrosis independently of perforin [66]. Increased activity of GzmB was noted in fibrotic expansion of human heart failure and murine models of heart failure (HF) induced by AngII. A deficiency in GzmB in this murine model showed protection against both fibrosis and hypertrophy [66].

A relatively unique finding in the top 10 IPA pathways in EN was an upregulation of eukaryotic elongation factor 2 (EIF2). This finding agrees with a report by Dieseldorff Jones et al. [67] demonstrating an increase in EIF2 signaling in HCM mice harboring a cTnC-A8V mutation. EIF2 and its phosphorylation product are implicated in HF-induced endoplasmic reticulum stress, as reported by George et al. [68]. Apart from implications that endoplasmic reticulum stress is related to pathological progression in various forms of cardiac dysfunction, it has also been demonstrated to be associated with cardiac hypertrophy induced by constant chronic hypoxia in mice [69]. As pointed out by Dieseldorff Jones et al. [67], the elevations in EIF2 may be related to the demonstration that genome-wide alterations in start-site usage in HCM promote EIF2 pathway enhancement [70]. Thus, our report supports that EIF2 is a significant factor in the general pathological process of EIF2 in cardiac dysfunction.

### 3.5 Comparison with human HCM septal samples, non-sarcomeric HCM, and other large animal models

A comparison of the pathways derived from the analysis reported here with the proteomic analysis of human septal samples provides insights into the differences and similarities in the pig heart model. An issue with this comparison is the wide diversity of proteomic approaches and analyses used. For example, a study by Tonry et al. [23] reported only 8 % overlap with the findings of Chen et al. [71], who reported a significant modification in cytoskeletal proteins, as we have reported in the porcine model. However, Tonry et al. [23] reported unique proteomic changes in obstructive HCM septal samples compared to other HF that agree with our findings. These included upregulation of proteins regulating fibrosis and transmembrane signaling involving matrix proteins, and MYH6, which has related pathways including cytoskeleton remodeling and regulation of the actin cytoskeleton by Rho GTPases [71]. Garmany et al. [52,72] reported results of analysis of human septal samples compared with controls using the Gene Ontology (GO) pathway. Some of this analysis coincided with our findings in pig hearts about the roles of Rho A signaling, integrin signaling, ILK signaling, and actin cytoskeletal signaling. Previs et al. reported an increase in abundance of z-disk signaling proteins and intermediate filaments in septal samples, which fits with our findings [9]. In contrast to the pig heart data, significant increases in PKA signaling and DCM signaling were reported in septal samples [73]. There were also adverse effects on metabolic signaling with depressed oxidative phosphorylation in the septal samples, which we found in the endocardium but not in the LV free wall.

In felines, which have a high incidence of HCM and represent another large animal model, proteomics has been reported for advanced pathology [74]. In agreement with the findings here in pig LV MYH7-R403Q variant samples, pathway analysis reported in feline models was related to mechano-signaling in the extracellular matrix, adhesion, immune responses, and fibrosis. Metabolic remodeling was also noted in the feline LV samples, unlike our findings with the LV free wall samples from the mini-pig model. However, the feline study did not separate the endocardium from the LV, as we did, so it is unclear if there were regional differences. In summary, there may be differences in the metabolic remodeling that are localized to the endocardium in the mini-pig heart. This may reflect a difference in the mechano-sensitivity of the endocardium in the mini-pig model.

In the non-sarcomeric HCM pediatric population, the incidence of multiple abnormalities and syndromes is 35 % of the patient population [75]. Pathways in both adult and pediatric non-sarcomeric HCM include mechano-energetic uncoupling, fibrosis signaling, inflammatory signaling, EIF2 signaling, and Rho-GDI signaling [[76], [77], [78], [79]]. This overlap of our findings with other stress-induced hypertrophy emphasizes the point of our investigation that in sarcomere mutation-linked HCM, there are pathological processes beyond the hypercontractility that need to be considered in therapeutic approaches. The mechano-energetic uncoupling will likely promote fibrosis, inflammatory/immune signaling, and altered mitochondrial metabolism [[41], [42], [43]]. These results support the main conclusion of our study of the porcine MYH7-R403Q variant model of interrelated signaling in inflammatory, mechanical, and fibrotic signaling. They also indicate a need for a more in-depth study of the mechanisms in HCM employing approaches that interrogate the myocyte microenvironment.

Metabolomic studies of human HCM septal samples have confirmed a severe adverse late-stage metabolic remodeling [9,73,80]. Earlier studies of heart samples from ∼1-year-old controls and Landrace pig hearts with naturally occurring hypertrophic cardiomyopathy (HCM) demonstrated depression in cytochrome oxidases and NADH dehydrogenase and a significant loss of mitochondrial DNA [81]. However, there was no investigation of the localization of the pathology. Mitochondrial alterations, which were identified in our study as associated with EN samples, confirm the earlier studies reporting that both energy supply and energy consumption are altered in HCM [10]. Our results and analytical approaches also support conclusions from earlier studies that included regional differences in transcriptional modifications and determinations of biomarkers prior to end-stage diseases [82]. Laird et al. [82] interrogated focal and spatially restricted alterations in the transcriptional pathways of areas of septal samples from HCM patients exhibiting normal and severely abnormal disarray. As in our data, their findings identified differences within the HCM septal samples for mitochondrial and extracellular matrix gene expression. Similar but less significant findings were reported in comparisons of control and HCM areas that did not display disarray. A related study performed respirometry on myectomy samples from HCM patients permeabilized with saponin and reported a decrease in oxidative phosphorylation that was strongly correlated with the abnormal organization of mitochondria [80]. Abundance and fragmentation of the mitochondria were not altered, but there was a disturbance in the organization of mitochondria in relation to the myofibrils. This depression of mitochondrial function and septal thickness was correlated with a depression in NADH-linked respiration. In contrast to this finding, an extensive omics analysis of metabolic pathways in myectomy samples from HCM patients reported multiple disturbances in energy-related biochemical pathways together with a depression of mitochondrial genes regulating creatine kinase and ATP synthesis [10]. Moreover, this study showed extreme damage to mitochondrial structure. These conflicting data regarding pathological mechanisms suggest a need to determine mitochondrial dysfunction over time with large animal models or hiPSC-CMs.

## 4 Implications for therapeutic approaches

Our findings of altered fibrotic signaling have been previously reported as a common disorder in stressed hearts and as a target for therapy. Challenges to direct inhibition of TGF-β have been noted [83], whereas interference of TGF-β signaling may be more amenable to modification by small molecules. One approach suggested has been the modulation of integrin-dependent TGF-β signaling [83,84]. There has been an extensive effort to precisely inhibit the subset of integrins αVβ1 and αVβ6 that have a role in TGF-β-induced fibrosis. However, no agents have been developed. Breakthrough findings may reside in identifying specific and unique cell populations of fibroblasts. One representative of this premise is the report of Chaffin et al. [5], who discovered a population of activated fibroblasts unique to HCM hearts using single-nucleus transcriptional profiling. Using genetic approaches, these investigators could show a reduction in the effects of TGF-β induction on myofibroblast state transitions. Along these lines, Bretherton et al. [85] reported expansion of a fibroblast population in DCM that promoted fibrosis that could be blocked by inhibiting p38 MAPK in fibroblasts. With earlier evidence of a convergence between fibrotic signaling in HCM and DCM, it is apparent that this approach may be therapeutic in HCM [5].

Several pathways in the top 10 of both EN and LV MYH7-R403Q samples support the idea of considering adjuvant therapies altering back-and-forth signaling to and from myocytes and ECM. Modifications in actin cytoskeleton signaling in both LV and EN heart samples support a significant role for inside/out and outside/in mechano-signaling in the context of the hypercontractility induced by the R403Q mutation.

In relation to our identification of ER signaling in the porcine HCM model, it is of interest that the signaling pathway involves modulation of apelin signaling via its GPCR, apelin receptor, which is druggable, as reviewed by Chapman et al. [86]. A recent Phase 1b human trial has followed pre-clinical studies reporting beneficial effects of AMG 986, an apelin receptor agonist, demonstrated to improve cardiac function independently of adverse effects on hemodynamics [87]. The effects of the compound in patients with HF await further studies.

In the case of mitochondrial dysfunction, which was noted in our study and others, there is evidence that increasing NADH-linked respiration was effective in improving oxidative phosphorylation [80]. Stabilizing cardiolipin with elamipretide and raising mitochondrial NAD+ levels both boosted NADH-linked respiration. Thus, our data and many others support the pursuit of discovering adjuvant therapies in HCM beyond reducing myofilament tension and myocardial wall stress.

## 5 Limitations and conclusions

Conclusions from our study are made with recognition of some limitations. One limitation is the lack of corroborating physiological and histological data providing a clear correlation with the phenotype. These studies with the MYH7-R403 pig model at the age used in our investigation remain for future validation. Moreover, we emphasize that the current study is a research article rather than a clinically relevant preclinical dataset. Despite these limitations, our proteomic analysis of the translationally relevant GE mini-pig model of a MYH7-R403Q variant revealed the importance of the micro-environment, where we found alterations in mechano-signaling involving compromised cell-to-cell interactions via the extracellular matrix. The global effect of the MYH7-R403Q variant in our studied mini-pig model was the activation of inflammation, offering a potential global therapeutic target. The disparate mitochondrial dysfunction in the endocardial tissue samples, supported by inhibition of oxidative phosphorylation, TCA cycle II, and estrogen receptor signaling, may suggest regional energetic therapeutic targeting. We highlight several alternative approaches for targeting novel pathways associated with the MYH7-R403Q mutation.

The following are the supplementary data related to this article.

Supplementary Table S1 Unmodified total proteins. Supplementary Table S1

Supplementary Table S2 Phospho-peptides. Supplementary Table S2

Supplementary material Image 1

## CRediT authorship contribution statement

**Chad M. Warren:** Writing – review & editing, Writing – original draft, Visualization, Project administration, Methodology, Investigation, Formal analysis, Conceptualization. **David M. Ryba:** Writing – review & editing, Resources, Methodology, Investigation, Funding acquisition, Conceptualization. **Gail E. Geist:** Writing – review & editing, Resources, Methodology, Investigation. **Aileen Castro Coronado:** Writing – review & editing, Resources, Methodology, Investigation. **Beata M. Wolska:** Writing – review & editing, Resources, Project administration, Funding acquisition. **Paul H. Goldspink:** Writing – review & editing, Resources, Project administration, Funding acquisition. **R. John Solaro:** Writing – review & editing, Writing – original draft, Resources, Project administration, Funding acquisition, Conceptualization.

## Declaration of competing interest

The authors declare the following financial interests/personal relationships, which may be considered as potential competing interests: RJS is on the scientific advisory board of CardiaTec Biosciences. DMR, GEG, and ACC are employees of Bristol Myers Squibb. If there are other authors, they declare that they have no known competing financial interests or personal relationships that could have appeared to influence the work reported in this paper.

## Data Availability

The proteomics dataset is available via the MassIVE data repository (MSV000097929), a full member of the Proteome Xchange Consortium (PXD064101). To access the MassIVE dataset using your preferred FTP client program (Filezilla, WinSCP, etc.), use ftp://massive-ftp.ucsd.edu/v09/MSV000097929/. All other data are contained within this published article (supplementary files).
