# Supplementary material for: Signaling pathway alterations in hearts of a porcine model harboring a β-myosin heavy chain (MYH7-R403Q) gene variant
Source: J Mol Cell Cardiol Plus. 2025 Nov 4;14:100495. doi: 10.1016/j.jmccpl.2025.100495 (PMC12639468; doi:10.1016/j.jmccpl.2025.100495)
Supplement: Supplementary file 3 — Supplementary material [file mmc3.docx]

Signaling Pathway Alterations in Hearts of a Porcine Model Harboring a β-Myosin Heavy Chain (MYH7-R403Q) Gene Variant

Chad M. Warren^1*^, David M. Ryba^2^, Gail E. Geist^2^, Aileen Castro Coronado^2^, Beata M. Wolska^1,3^, Paul H. Goldspink^1^, R. John Solaro^1^

^1^Department of Physiology and Biophysics, Center for Cardiovascular Research, University of Illinois Chicago ^2^Bristol Myers Squibb, Brisbane, California, USA ^3^Department of Medicine, Division of Cardiology, University of Illinois Chicago

**Supplementary methods**

**2.3 Immunoblotting**

A portion of the whole homogenate samples prepared for the phospho-proteomic analysis were diluted at least 1:1 with industrial-strength sample buffer (ISB: 8 M urea, 2 M thiourea, 50 mM Tris pH 6.8, 3% w/v SDS, 75 mM DTT, and 0.05% w/v bromophenol blue) [1] to 1.25 mg/ml. The protein concentration was determined with the Pierce 660 nm protein assay (#22660), including the ionic detergent compatibility reagent (#22663), following the manufacturer’s protocol. The samples (12.5-15 µg/lane) were loaded on 12 or 15% (w/v) total acrylamide SDS-PAGE gels, with 0.5% (w/v) bis-acrylamide for the resolving gel as previously described [1]. The gels were cast in a BioRad Criterion empty cassette and then electrophoretically separated in a BioRad Criterion cell at 200V constant voltage for 75 min in 25 mM Tris base, 192 mM Glycine, and 0.1% (v/v) SDS at room temperature. The proteins from the gel were transferred onto a 0.2 µm polyvinylidene difluoride (PVDF) membrane with a BioRad Criterion blotter in 10 mM CAPS, pH 11.0 [2] without methanol at 25 or 30V for 90 min, chilled with a blue ice pack.

After the transfer, the membranes were stained for all proteins with Swift^TM^ stain (G Biosciences #786-677) following the manufacturer’s recommendations to serve as a loading control instead of using a housekeeping protein [3, 4]. The membranes were blocked with 5% (w/v) non-fat milk in 50 mM Tris-HCl, pH 7.6, 200mM NaCl with 0.1% (v/v) Tween-20 (TBST). The immunoblots were incubated with primary antibodies overnight at 4°C, washed with TBST, incubated with secondary antibodies for 90 min at room temperature, and washed again with TBST. Immunoblots that were probed for more than one target were incubated with 30% (v/v) H2O2 for 30 min at room temperature to inactivate the prior secondary HRP-conjugated antibody, then the immunoblot was washed with TBST and incubated with the next primary antibody from a different host [5]. The primary antibodies used were: anti-vinculin 1:2000, Sigma #V4505; anti-cytochrome c oxidase 4 isoform 2 (COX4I2) 1:1000. Proteintech #11463-1-AP; anti-sarcoplasmic/endoplasmic reticulum Ca^2+^ ATPase 2a (SERCA2a) 1:20,000, Badrilla #A010-23AP; anti-alpha-actinin 1:2000, Abcam #ab9465; anti-camitine palmitoyltransferases 1B (CPT1B) 1:10,000, Proteintech #22170-1-AP; anti-supervillin 1:500, Proteintech #27524-1-AP; and all were diluted in 5% milk in TBST except supervillin, which was diluted in 1% (w/v) BSA in TBST. The secondary antibodies used were: goat anti-rabbit-HRP 1:20,000, Cell Signaling Technologies #7074; goat anti-mouse-HRP 1:20,000, Cell Signaling Technologies #7076; mouse anti-rabbit-HRP conformational specific 1:20,000, Cell Signaling Technologies #5127 which was only used with the supervillin antibody. All secondary antibodies were diluted in 5% (w/v) milk in TBST.

The immunoblots were developed with SuperSignal West Femto substrate (Thermo #34096) following the manufacturer’s recommendations and imaged with a BioRad Chemidoc MP. The images were densitometrically analyzed with BioRad’s ImageLab v. 6.1 and Microsoft Excel 360. The data were statistically analyzed and graphed with GraphPad Prism v. 10.5.

**Protein S-TRAP digestion and TMTpro labeling**

Protein samples (200 µg) were digested with an S-Trap mini ([www.protifi.com](http://www.protifi.com)) following the manufacturer’s recommendations with modifications. The samples were treated with benzonase (0.68 units/µg of protein; Millipore # 70664-3) for 15 min at room temperature and then alkylated with a final iodoacetamide concentration of 50mM for 45 min at room temperature. To quench the alkylation reaction, 33 mM DTT was added and incubated for 20 min at room temperature. Next, the samples were acidified, and binding/wash buffer (100 mM TEAB, pH 8.5, 90% methanol) was added to the sample for binding to the S-Trap as recommended by the manufacturer. The S-Trap column was washed five times with binding/wash buffer and then 125 µL of digestion buffer (100 mM TEAB, pH 8.5) with 1:10 Trypsin Platinum (Promega #VA9000) and 1:50 Lys-C (Worthington Biochemical Corp. #LS02144) protein: enzyme ratio was added to the column for digestion. The digestion was done in a humid chamber at 37ºC for 1.5 hours, and then an additional 50 µL of digestion buffer was added and incubated overnight. The digested protein peptides were eluted with 1) 80 µL of 50 mM TEAB pH 8.5, 2) 80 µL of 0.2% (v/v) formic acid 3 times, 3) 80 µL of 50 (v/v) acetonitrile, 0.2% (v/v) formic acid, and all elutions were combined and speed vac to dryness and stored at -80ºC.

The pooled peptides of each sample were resuspended in 50 µL of 8M Urea, 100mM TEAB, pH 8.5, and the peptide concentration was determined using Pierce’s Fluorometric peptide assay (#23290) following the manufacturer’s recommendations. All peptide samples were diluted to the same concentration, and we removed ~6.25% (v/v) from each sample and equally mixed them for an internal global standard. The peptide samples (100 µg each) were randomly isobarically labeled to help control for labeling bias with Thermo FisherScientific’s TMTpro (Tandem Mass Tag) 16-plex reagents (cat# A44521; lot# XB339670) following the manufacturer’s recommendations. The specific TMT channels used for each sample are listed in the header of the supplementary Table S1. The labeled and quenched samples were then equally mixed into two separate experiments (1+2) due to the number of samples. The internal global standard was labeled with the TMT126-channel for both experiments. The two separate experiments were speedvac down to dryness and stored at -80°C.

**2.5 Phospho-peptide enrichment and high pH reverse-phase fractionation**

The two TMTpro labeled experiments were resuspended in 300 µL of 3% (v/v) trifluoracetic acid to do peptide desalting chromatography before phospho-enrichment. The Pierce peptide desalting spin columns (#89851) were first used as directed by the manufacturer. The sample flow through and first wash from the peptide desalting column was saved and applied serially to a Graphite spin column (Pierce #88302) following the manufacturer’s recommendations to further capture and desalt peptides. The elutions from both columns were then combined and speedvac down to dryness. The dried peptide was resuspended in 100 µL of 25% (v/v) acetonitrile, 0.1%TFA (v/v), and a colorimetric peptide assay (ThermoScientific #23275) was performed as suggested by the manufacturer to determine peptide concentration and separate into two aliquots containing 5% and 95% of the total peptides. The 5% aliquot was saved for non-enriched peptides, also known as the “total”, and speed vac to dryness. The 95 % aliquot was saved for phosphopeptide enrichment by Sequential enrichment of Metal Oxide Affinity Chromatography (High-Select^TM^ SMOAC) (ThermoScientific # A32993 and A32992) following the manufacturer’s recommendations. The phosphopeptide enrichment was first done with TiO2, and then Fe-NTA was serially performed as suggested by the manufacturer. The eluted phosphopeptides were combined and speed vac to dryness and stored at -80°C [6]. To fractionate both the non-enriched and phosphopeptide enriched samples, we used a Pierce High pH Reversed-Phase peptide fractionation kit (Cat# 84868) with modifications as previously described [6] except the final elution was done with 50% acetonitrile. The fractions were pooled to 6-8 and used to acquire mass spectrometric data.

**2.6 Mass spectrometric data acquisition**

The peptide fractions were resuspended in 3% (v/v) acetonitrile and 0.1% (v/v) formic acid. 800 ng of each peptide fraction was injected in 6 µl sequentially with an Ultimate 3000 RSLCnano UHPLC in conjunction with an EASY-Spray source operating in positive ion mode into the Thermo Scientific Orbitrap Fusion Lumos. A Thermo Scientific Acclaim PepMap 100 C18 reversed-phase column (DX164199, 100 µm X 20 mm, 100 Å, 5 µm) trapped the peptides, and the separating column was a C18 reverse-phase Thermo Scientific column (ES802, 75 µm X 250mm, 100 Å, 2µm). The peptides were eluted with an acetonitrile gradient from 3-55% over 101 min with a flow rate of 500 nl/min at 40ºC. The Orbitrap Fusion Lumos parameters were as previously described with minor modifications[6, 7]. An Orbitrap survey scan was done from 400-1600 m/z with a resolution of 120K, a target of 4 X 10^5^ ions, or a maximum injection time of 50 ms. The linear ion trap in turbo mode was used to acquire data-dependent MS/MS spectra with collision-induced dissociation at 35% energy and an activation Q=0.25. The MS/MS spectra were acquired with a target of 1 X 10^4^ ions or a maximum of 35 ms, an isolation mass window of 0.7 m/z, charge states 2-6, and a 45s dynamic exclusion. Synchronous precursor selection with up to 10 precursors was selected for MS3 with higher-energy collisional dissociation at 55% energy, a mass range of 100-500 m/z, resolution of 50,000, and a target of 1 X 10^5^ ions or a maximum injection time of 200 ms. A precursor selection range of 400-1600 m/z was used, along with an isolation window of 2 m/z and a fixed cycle time of 3 s.

**2.7 Mass spectrometric database alignment and TMT quantification**

Thermo .raw data files were obtained from the UC-Davis Proteomics core, and we then searched the data using PEAKS Studio v10.6 build 20201221 (Bioinformatics Solutions Inc.). The database search parameters were precursor mass error of 15 ppm, fragment mass error of 0.5 Da, and the enzyme was specific trypsin with a maximum of two missed cleavages. One fixed modification of TMT (Tandem Mass Tag) 16plex +304.21 and up to four variable modifications: carbamidomethylation (C) +57.02, deamidation (NQ) +0.98, oxidation (M) +15.99, phosphorylation (STY) +79.97, with a maximum of 5 variable modifications per peptide. The database alignment was to a combined Sus Scrofa non-reviewed and reviewed TrEMBL UniProtKB Swiss-Prot (UP000008227) downloaded on Jan 1st, 2023, with 46,255 entries in a decoy-fusion approach allowing for a more conservative false discovery rate (FDR) estimation [8]. The combined database was used due to poor annotation in the reviewed Sus Scrofa database. A contaminant database cRAP downloaded March 4^th^, 2019, from [www.thegpm.org/crap/](http://www.thegpm.org/crap/) was used for a contaminant database. A peptide FDR cutoff of 1%, and ≥ 2 unique peptides were required for positive protein identification. The precursor mass correction and charge 2-7 filter were true for data refinement. Phosphorylation-specific analysis was done at the peptide level; thus, the filter criteria were altered to require ≥ 1 unique peptide for positive protein identification. In addition, an A-score > 13 was required for inclusion and considered confidently localized to an amino acid [9].

The initial TMT quantification was first done in PEAKS Studio v10.6 build 20201221 (Bioinformatics Solutions Inc.). Then, the normalized data were exported to an Excel file, which was then statistically analyzed by GraphPad Prism 9.5.1 and OriginPro 2023b (64-bit). The initial PEAKS Studio analysis was done with a mass error of 15 ppm on the MS3 reporter ion with purity correction. Both intra- and inter-normalization were done utilizing the total ion counts method when both experiments were analyzed in the case of the EN group comparisons; however, in the LV and CA comparisons, only the intra-normalization was done. The inter-normalization used the global internal standard to link the two TMT-labeled experiments using the TMT channel 126. In each binary comparison (R403Q *vs.* WT), a reference label was assigned in PEAKS Studio for intra-auto-normalization and ratio purposes. The reference label was chosen based on the WT control group for the binary comparison and the TMT channel with the most identifications. The reference channel proteins were required for comparison. The normalized data were then exported to an Excel document for further analysis, as described in the statistical analysis section.

**Supplemental Figure 1.**

**
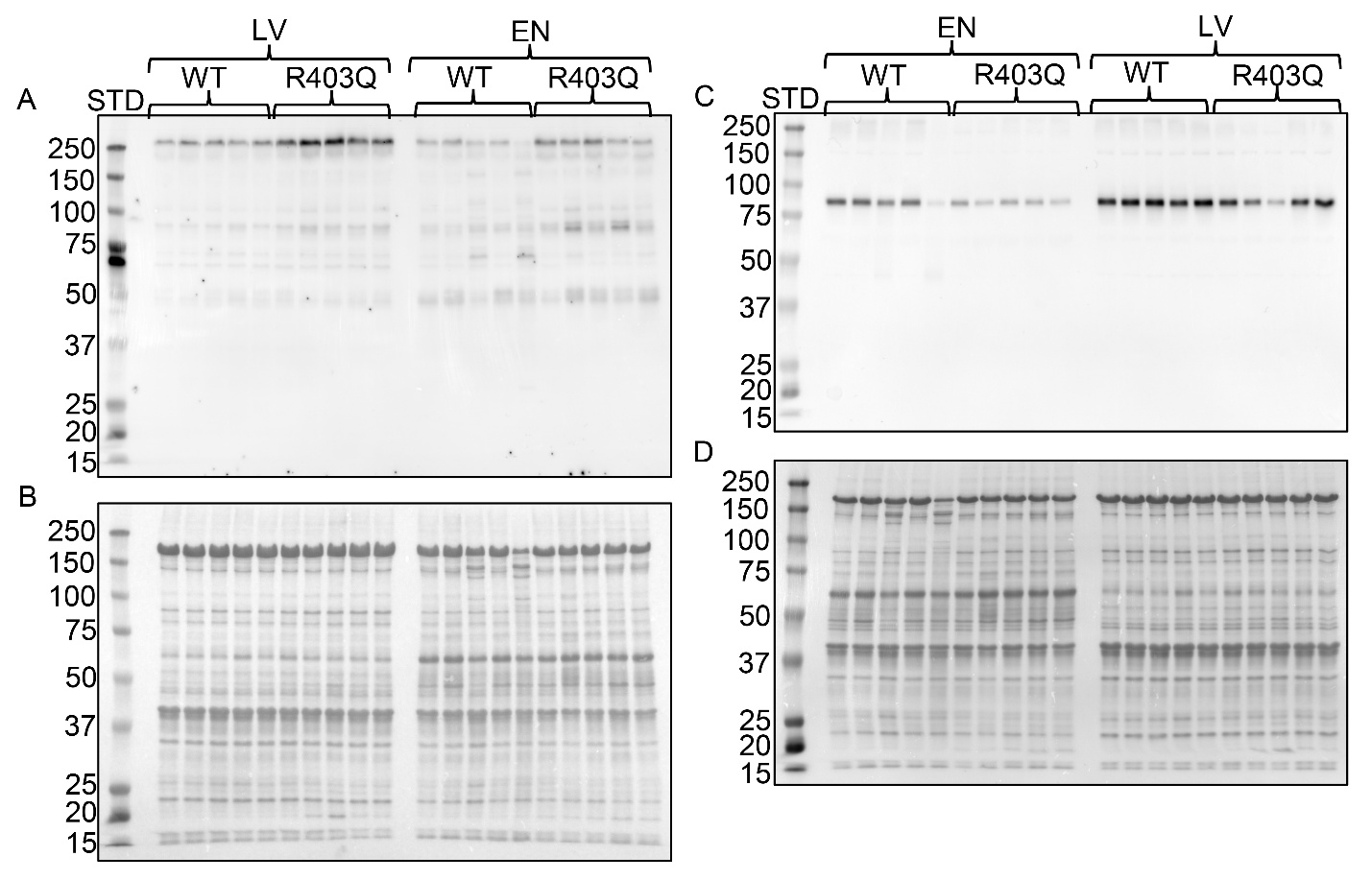
**

**Supplemental Figure 1.** The original uncropped images used for Figure 2. **A.** Colorimetric and Chemi image merged to show the bands detected along with the molecular weight marker for Fig. 2A upper image. The Chemi image was used for densitometry. **B.** Colorimetric image of the Swift-stained membrane used for the lower image of Fig. 2A. The lane identifications are the same as Panel A above. Images A and B are from the same membrane. **C.** Colorimetric and Chemi image merged to show the bands detected along with the molecular weight marker for Fig. 2B upper image. The Chemi image was used for densitometry. **D.** Colorimetric image of the Swift-stained membrane used for the lower image of Fig. 2B. The lane identifications are the same as Panel C above. Images C and D are from the same membrane. STD, molecular weight standard; LV, left ventricle; EN, endocardium; WT, wildtype; R403Q, MYH7-R403Q mutant. The chemiluminescent signals were produced by HRP-conjugated secondary antibodies exposed to SuperSignal West Femto (Thermo #34096) substrate. All images were captured with a BioRad Chemidoc MP and exported as TIFF images for publication. The densitometric analysis was done with BioRad Image Lab v. 6.1. Note: The image in panel D is the same as Supplementary Fig.5B because the same membrane was used to probe multiple targets.

**Supplementary Fig. 2**

**
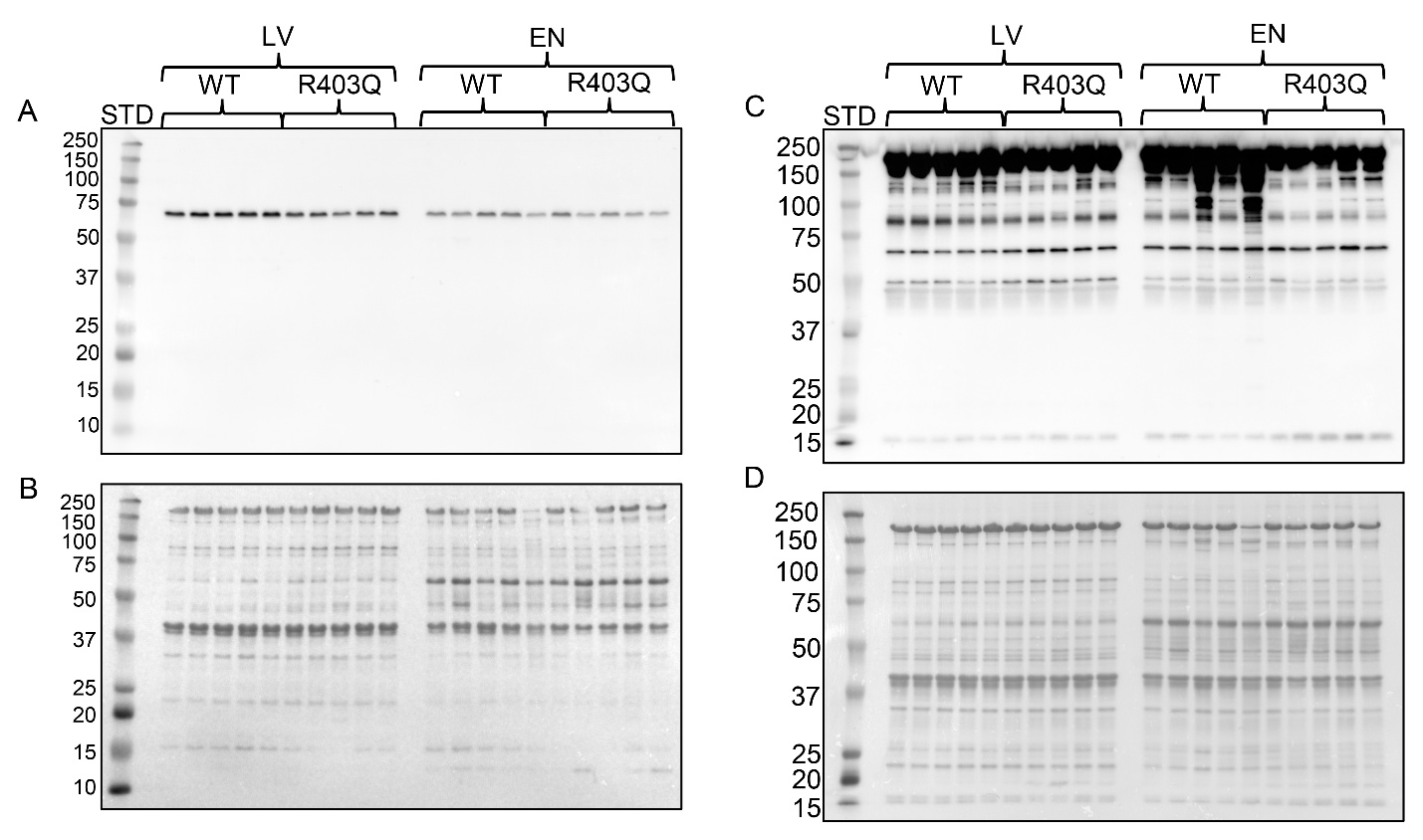
**

**Supplemental Figure 2.** The original uncropped images used for Figure 3. A. Colorimetric and Chemi image merged to show the bands detected along with the molecular weight marker for Fig. 3A upper image. The Chemi image was used for densitometry. B. Colorimetric image of the Swift-stained membrane used for the lower image of Fig. 3A. The lane identifications are the same as Panel A above. Images A and B are from the same membrane. C. Colorimetric and Chemi image merged to show the bands detected along with the molecular weight marker for Fig. 3B upper image. The Chemi image was used for densitometry. D. Colorimetric image of the Swift-stained membrane used for the lower image of Fig. 3B. The lane identifications are the same as Panel C above. Images C and D are from the same membrane. STD, molecular weight standard; LV, left ventricle; EN, endocardium; WT, wildtype; R403Q, MYH7-R403Q mutant. The chemiluminescent signals were produced by HRP-conjugated secondary antibodies exposed to SuperSignal West Femto (Thermo #34096) substrate. All images were captured with a BioRad Chemidoc MP and exported as TIFF images for publication. The densitometric analysis was done with BioRad Image Lab v. 6.1. Note: The image in panel D is the same as Supplementary Fig.5D because the same membrane was used to probe multiple targets.

**Supplementary Figure 3**

**
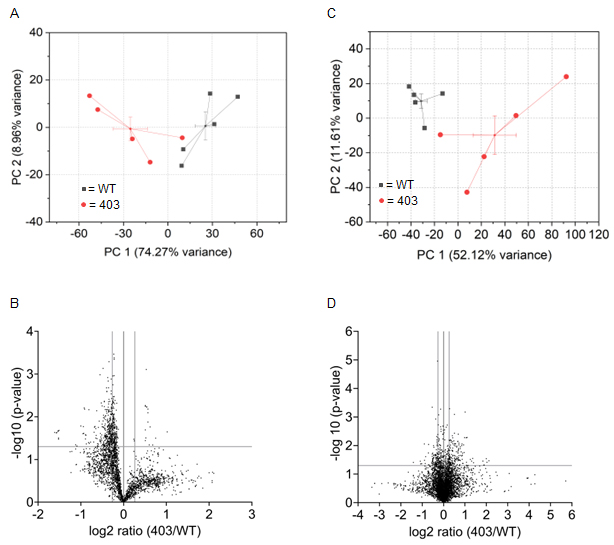
**

**Supplemental Figure 3.** Phospho-peptide and total unmodified protein analysis of data distribution and differences in coronary arteries (CA) samples. **A.** Principal component (PC) analysis of total unmodified proteins of CA shows two distinct populations for the wildtype (WT) compared to the MYH7-R403Q mutant (403). Error bars represent standard error, n=5. **B.** Volcano plot of differential protein abundances from total unmodified proteins of CA between the 403) and WT, plotting -log10 p-values and log2 ratio, n=5. Vertical gray lines = ≥ ±20% difference; horizontal gray line multiple unpaired t-test <0.05 p-value. Note: no points have a q-value < 0.05 (FDR) based on multiple comparison corrections with a two-stage step-up (Benjamini, Krieger, and Yekutieli). **C.** PC analysis of phospho-peptides of CA shows two distinct populations for the WT compared to the 403. Error bars represent standard error, n=5. **D.** Volcano plot of differential protein abundances from phospho-peptides of CA between the 403 and WT, plotting -log10 p-values and log2 ratio, n=5. Vertical gray lines = ≥ ±20% difference; horizontal gray line multiple unpaired t-test <0.05 p-value. Note: no points have a q-value < 0.05 (FDR) based on multiple comparison corrections with a two-stage step-up (Benjamini, Krieger, and Yekutieli).

**Supplemental Figure 4**

**
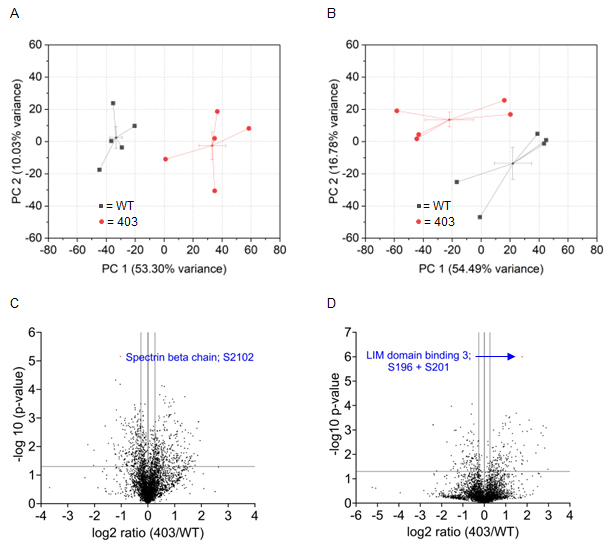
**

**Supplemental Figure 4.** Phospho-peptide analysis of data distribution and differences in left ventricle free-wall (LV) and endocardium (EN) samples. **A.** Principal component (PC) analysis of phospho-peptides of LV shows two distinct populations for the wild type (WT) compared to the MYH7-R403Q mutant (403). Error bars represent standard error, n=5. **B.** PC analysis of phospho-peptide EN shows two distinct populations for the WT compared to 403. Error bars represent standard error, n=5. **C.** Volcano plot of differential protein abundances from phospho-peptides of LV between the 403 and WT, plotting -log10 p-values and log2 ratio, n=5. Vertical gray lines = ≥ ±20% difference; horizontal gray line multiple unpaired t-test <0.05 p-value. Note: The red point has a q-value < 0.05 (FDR) based on multiple comparison corrections with a two-stage step-up (Benjamini, Krieger, and Yekutieli). **D.** Volcano plot of differential protein abundances from phospho-peptides of EN between the 403 and WT, plotting -log10 p-values and log2 ratio, n=5. Vertical gray lines = ≥ ±20% difference; horizontal gray line multiple unpaired t-test <0.05 p-value. Note: the red point has a q-value < 0.05 (FDR) based on multiple comparison corrections with a two-stage step-up (Benjamini, Krieger, and Yekutieli).

**Supplemental Figure 5.**

**
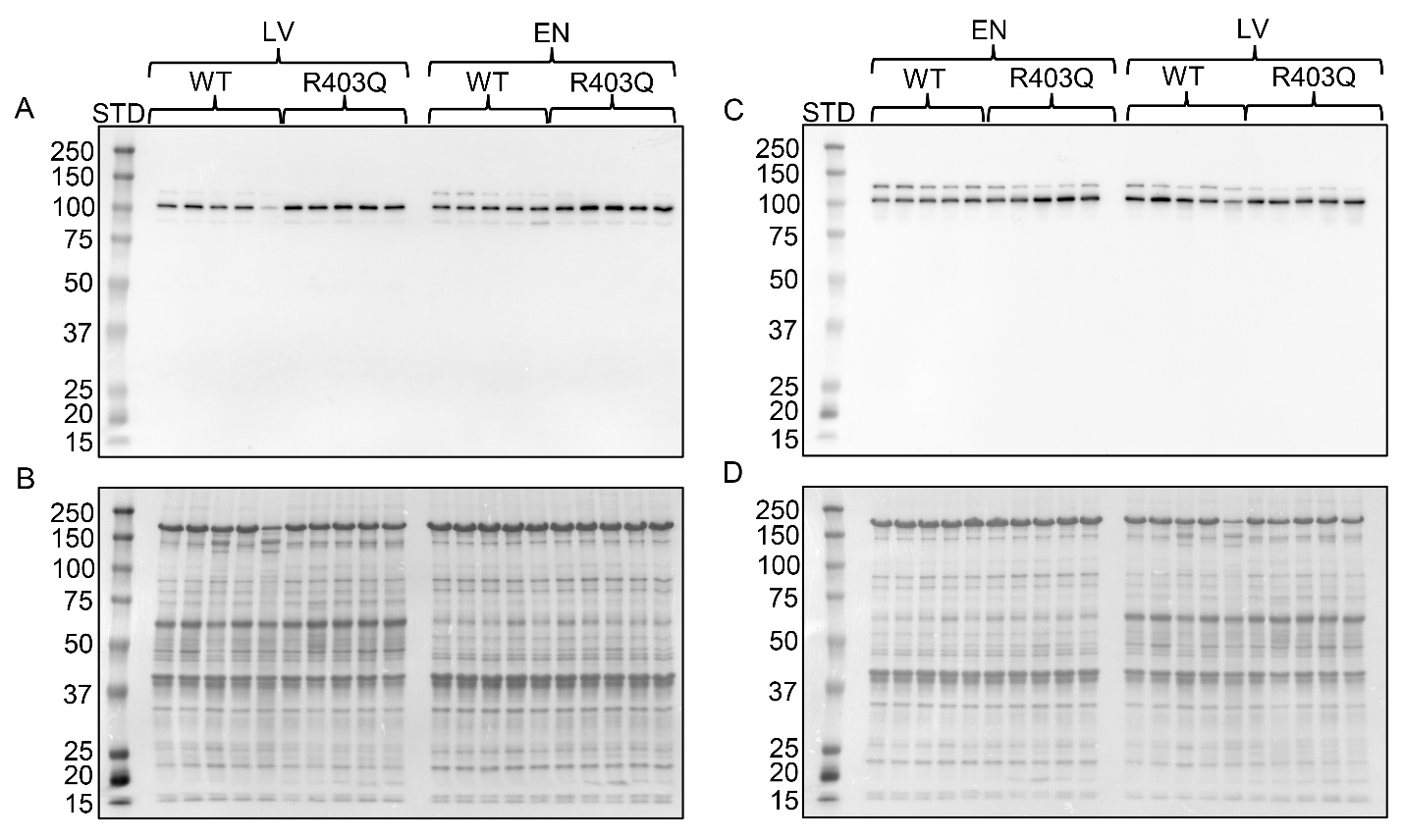
**

**Supplemental Figure 5.** The original uncropped images used for Figure 6. A. Colorimetric and Chemi image merged to show the bands detected along with the molecular weight marker for Fig. 6A upper image. The Chemi image was used for densitometry. B. Colorimetric image of the Swift-stained membrane used for the lower image of Fig. 6A. The lane identifications are the same as Panel A above. Images A and B are from the same membrane. C. Colorimetric and Chemi image merged to show the bands detected along with the molecular weight marker for Fig. 6B upper image. The Chemi image was used for densitometry. D. Colorimetric image of the Swift-stained membrane used for the lower image of Fig. 6B. The lane identifications are the same as Panel C above. Images C and D are from the same membrane. STD, molecular weight standard; LV, left ventricle; EN, endocardium; WT, wildtype; R403Q, MYH7-R403Q mutant. The chemiluminescent signals were produced by HRP-conjugated secondary antibodies exposed to SuperSignal West Femto (Thermo #34096) substrate. All images were captured with a BioRad Chemidoc MP and exported as TIFF images for publication. The densitometric analysis was done with BioRad Image Lab v. 6.1. Note: The images in panels B and D are the same as Supplementary Figs. 2D and 3D because the same membrane was used to probe multiple targets.

**References**

1. Fritz, J.D., D.R. Swartz, and M.L. Greaser, *Factors affecting polyacrylamide gel electrophoresis and electroblotting of high-molecular-weight myofibrillar proteins.* Anal Biochem, 1989. **180**(2): p. 205-10.

2. Matsudaira, P., *Sequence from picomole quantities of proteins electroblotted onto polyvinylidene difluoride membranes.* J Biol Chem, 1987. **262**(21): p. 10035-8.

3. Aldridge, G.M., et al., *The use of total protein stains as loading controls: an alternative to high-abundance single-protein controls in semi-quantitative immunoblotting.* J Neurosci Methods, 2008. **172**(2): p. 250-4.

4. Van Ry, P.M., et al., *Laminin-111 improves muscle repair in a mouse model of merosin-deficient congenital muscular dystrophy.* Hum Mol Genet, 2014. **23**(2): p. 383-96.

5. Sennepin, A.D., et al., *Multiple reprobing of Western blots after inactivation of peroxidase activity by its substrate, hydrogen peroxide.* Anal Biochem, 2009. **393**(1): p. 129-31.

6. Warren, C.M., et al., *Truncation of the N-terminus of cardiac troponin I initiates adaptive remodeling of the myocardial proteosome via phosphorylation of mechano-sensitive signaling pathways.* Mol Cell Biochem, 2022. **477**(6): p. 1803-1815.

7. Erickson, B.K., et al., *Evaluating multiplexed quantitative phosphopeptide analysis on a hybrid quadrupole mass filter/linear ion trap/orbitrap mass spectrometer.* Anal Chem, 2015. **87**(2): p. 1241-9.

8. Zhang, J., et al., *PEAKS DB: de novo sequencing assisted database search for sensitive and accurate peptide identification.* Mol Cell Proteomics, 2012. **11**(4): p. M111 010587.

9. Jedrychowski, M.P., et al., *Evaluation of HCD- and CID-type fragmentation within their respective detection platforms for murine phosphoproteomics.* Mol Cell Proteomics, 2011. **10**(12): p. M111 009910.
